# Influence of acute pancreatitis on the in vitro responsiveness of rat mesenteric and pulmonary arteries

**DOI:** 10.1186/1471-230X-8-19

**Published:** 2008-05-29

**Authors:** Enilton A Camargo, Maria Andréia Delbin, Tatiane Ferreira, Elen CT Landucci, Edson Antunes, Angelina Zanesco

**Affiliations:** 1 Department of Pharmacology, Faculty of Medical Sciences, P.O. Box 6111, State University of Campinas (UNICAMP), Campinas (SP), Brazil; 2 Department of Physical Education, Institute of Biosciences, University of São Paulo State (UNESP), Rio Claro (SP), Brazil

## Abstract

**Background:**

Acute pancreatitis is an inflammatory disease characterized by local tissue injury and systemic inflammatory response leading to massive nitric oxide (NO) production and haemodynamic disturbances. Therefore, the aim of this work was to evaluate the vascular reactivity of pulmonary and mesenteric artery rings from rats submitted to experimental pancreatitis.

Male Wistar rats were divided into three groups: saline (SAL); tauracholate (TAU) and phospholipase A_2 _(PLA_2_). Pancreatitis was induced by administration of TAU or PLA_2 _from *Naja mocambique mocambique *into the common bile duct of rats, and after 4 h of duct injection the animals were sacrificed. Concentration-response curves to acetylcholine (ACh), sodium nitroprusside (SNP) and phenylephrine (PHE) in isolated mesenteric and pulmonary arteries were obtained. Potency (pEC_50_) and maximal responses (E_MAX_) were determined. Blood samples were collected for biochemical analysis.

**Results:**

In mesenteric rings, the potency for ACh was significantly decreased from animals treated with TAU (about 4.2-fold) or PLA_2 _(about 6.9-fold) compared to saline group without changes in the maximal responses. Neither pEC_50 _nor E_MAX _values for Ach were altered in pulmonary rings in any group. Similarly, the pEC_50 _and the E_MAX _values for SNP were not changed in both preparations in any group. The potency for PHE was significantly decreased in rat mesenteric and pulmonary rings from TAU group compared to SAL group (about 2.2- and 2.69-fold, for mesenteric and pulmonary rings, respectively). No changes were seen in the E_MAX _for PHE. The nitrite/nitrate (NO_x_^-^) levels were markedly increased in animals submitted to acute pancreatitis as compared to SAL group, approximately 76 and 68% in TAU and PLA_2 _protocol, respectively.

**Conclusion:**

Acute pancreatitis provoked deleterious effects in endothelium-dependent relaxing response for ACh in mesenteric rings that were strongly associated with high plasma NO_x_^- ^levels as consequence of intense inflammatory responses. Furthermore, the subsensitivity of contractile response to PHE in both mesenteric and pulmonary rings might be due to the complications of this pathological condition in the early stage of pancreatitis.

## Background

Epidemiological studies have shown the incidence of acute pancreatitis is increasing in the Western world, varying from 5 to 80 cases per 100,000 habitants. The most common causes of acute pancreatitis include alcoholism and gallstones, and the impairment of lung function associated with vascular complications are the main causes of the mortality, particularly in the severe forms of this disease [1-4].

Acute pancreatitis is a disease characterized by activation of pancreatic enzymes, ranging from mild, self-limited type of edematous pancreatitis to severe necrotizing form. In the most severe form of the disease, it is observed tissue damage, local inflammatory reaction and haemorrhage that are associated with remote organ failure, sepsis, and a high prevalence of mortality [5]. The complications of acute pancreatitis are mainly associated with the impairment of the lung function, which closely resembles the adult respiratory distress syndrome associated with other pathological conditions such as shock and ischemia/reperfusion [4]. It is known that releasing of inflammatory mediators and activated leukocytes generated in the acute pancreatitis contribute to the tissue damage and multiple organ failure, but the pathophysiological events of acute pancreatitis are not fully understood [6-8].

The haemodynamic disturbances of acute pancreatic are characterized by a marked arterial hypotension that is strongly related to shock syndrome, reperfusion failure and high plasma nitric oxide levels [9]. However, no studies exist investigating the reactivity of vascular smooth muscle to relaxing and contracting agents in experimental model of pancreatitis. Therefore, the aim of this work was to evaluate the vascular reactivity of pulmonary and mesenteric artery rings from rats submitted to experimental acute pancreatitis. To achieve this, animals were submitted to either sodium taurocholate (TAU) or phospholipase A_2 _(PLA_2_; from *Naja mocambique mocambique *venom) in the common bile duct, the former of which cause a severe haemorragic necrotizing pancreatitis [10] whereas the latter causes an edematous form of pancreatitis [11,12].

## Methods

### Animals

All experiments were carried out in accordance with the guidelines for animal care of the State University of Campinas (UNICAMP). Animals were housed in the animal care facility at the Department of Pharmacology in a room maintained at 20–21°C with 12:12-h light-dark cycle. Male Wistar rats, weighing 230–280 g, were divided into three groups named: saline (SAL)-; taurocholate (TAU)- and phospholipase A_2 _(PLA_2_)-injected animals.

### Acute pancreatitis induction

Acute pancreatitis was induced by the injection of taurocholate or PLA_2 _from *Naja mocambique mocambique *into the common bile duct of rats, as described previously [17,20]. Briefly, the animals were anaesthetized with a mixture of ketamine (25 mg/kg, i.p) and xylazine (10 mg/kg, i.p) and a medium laparotomy was performed. The duodenal loop was exteriorized and the common bile duct was cannulated transduodenally with a polyethylene tube. Saline (0.9%; vehicle), sodium tauracholate (5%) or PLA_2 _(obtained from *Naja mocambique mocambique *venom; 300 μg/kg) were injected into the duct in a final volume of 0.3 ml, with a constant flow over a 1 min period. The hepatic portion of the biliopancreatic duct was clamped before injecting the solutions, after which the abdomen was closed in two layers. After 4 h of duct injection, the animals were sacrificed (ketamine/xylazine, i.p.) and blood samples were collected for biochemical analysis. Mesenteric and pulmonary artery rings were isolated for construction of concentration-response curves.

### Concentration-response curves to acetylcholine, sodium nitroprusside and phenylephrine

The mesenteric and pulmonary arteries were isolated carefully and placed in freshly prepared Krebs solution containing (mM): NaCl, 118: NaHCO_3_, 25; glucose, 5.6; KCl, 4.7; KH_2_PO_4_, 1.2; MgSO_4 _7H_2_O, 1.17 and Cacl_2 _2H_2_O, 2.5. The arteries were cleaned of all adherent tissue and cut in rings of approximately 2 mm. Each ring was suspended between two wire hooks and mounted in 10 ml organ chambers with Krebs solution at 37°C, pH 7.4 and continuously aerated with a mixture of 95% O_2 _and 5% CO_2 _under a resting tension of 10 mN. After 1 hour of stabilization period, the tissues were pre-contracted with KCl 80 mM and washed. Cumulative concentration-response curves to vasodilator agents: acetylcholine (ACh; 10 nM – 100 μM for both arteries) and sodium nitroprusside (SNP; 100 pM – 1 μM for mesenteric and 100 pM – 3 μM for pulmonary artery) were obtained. Relaxing responses were calculated relative to the maximal changes from the pre-contraction produced by phenylephrine in each preparation, which was taken as 100% (PHE, 1 μM for mesenteric rings and 10 μM for pulmonary artery rings).

Concentration-response curves were also obtained for the α-adrenergic agonist, phenylephrine (PHE; 1 nM – 10 μM for both arteries), in the presence of beta-blocker, propranolol (100 nM). The contraction responses were calculated relative to the maximal changes from pre-contraction produced by KCl 80 mM in each preparation, which was taken as 100%.

All concentration-response data were evaluated for a fit to a logistics function in the form:

E = Emax/((1 + (10^c^/10^x^)^n^) + Φ)

where E is the effect of above basal; Emax is the maximum response produced by the agonist; c is the logarithm of the EC_50_, the concentration of agonist that produces half-maximal response; x is the logarithm of the concentration of agonist; the exponential term, n is a curve-fitting parameter that defines the slope of the concentration response line, and Φ is the response observed in the absence of added agonist. Nonlinear regression analysis to determine the parameters Emax, log EC_50_, and n were done using GraphPad Prism (GraphPad Software Inc., San Diego, CA) with the constraint that Φ = 0. The responses for each agonist are showed as the mean ± SEM of pEC_50 _and E_MAX_.

### Determination of serum nitrite/nitrate (NO_x_^-^) levels

In order to evaluate the NO production, the serum levels of nitrite (NO_2_^-^) plus nitrate (NO_3_^-^) were measured. Briefly, immediately after collecting arterial blood, the samples were centrifuged (8,000 g) for 10 min, and the resulting serum supernatant was stored at -80°C. Serum samples were ultrafiltered through microfilter cups (Microcon Centrifugal Filter Units, 10 kDa; Millipore, Bedford, MA, USA). The NO_x_^- ^concentration of the resulting filtrate solution was determined using a commercially available kit (Cayman Chemical, Ann Arbor, MI, USA) according to the manufacturer's instructions. This assay determines the total NO based on the enzymatic conversion of nitrate to nitrite by nitrate reductase. After the conversion, the spectrophotometric measurement of nitrite is accomplished by using the Griess reaction. The resulting deep purple azo compound absorbs light at 540–550 nm.

### Drugs and solutions

Acetylcholine, PLA_2 _from *Naja mocambique mocambique *venom, sodium nitroprusside, sodium taurocholate (taurocholic acid and sodium salt) and phenylephrine were purchased from Sigma (St. Louis, MO, USA).

### Statistical analysis

Data are expressed as mean ± SEM of n experiments. Analysis of variance (ANOVA) for repeated measurements was performed for the appropriate results and Bonferroni method was chosen as a post-test. A p values smaller than 0.05 was considered statistically significant.

## Results

### PLA_2 _and TAU-induced rat pancreatitis

The injection of PLA_2 _(from *Naja mocambique mocambique *venom) in the common bile duct at 300 μg/kg (4 h) markedly increased the pancreatic plasma protein extravasation and neutrophil influx (as evaluated by the increased myeloperoxidase activity), which was accompanied by neutrophil accumulation into the lungs and elevated serum amylase levels (Table 1), thus reproducing an acute pancreatitis condition. Similarly, injection of TAU (5%, 4 h) in the common bile duct increased significantly all parameters tests in comparison with saline group (Table 1). In TAU group, the lung myeloperoxidase activity and serum amylase levels were significantly higher than that of PLA_2 _group (*P *< 0.05).

**Table 1:** Measurements of pancreatic plasma extravasation, pancreatic and lung myeloperoxidase (MPO) activity and serum amylase levels in phospholipase A_2_(PLA_2_) – and taurocholate (TAU)-induced acute pancreatitis in rats.

**Groups**	**Pancreatic plasma extravasation (μl of plasma)**	**Pancreatic MPO (U/mg of tissue)**	**Lung MPO (U/mg of tissue)**	**Serum Amylase (U/l)**
**SAL**	198 ± 10	1.4 ± 0.3	18 ± 1	408 ± 21
**PLA**_2_	303 ± 30**	4.3 ± 0.6**	32 ± 3*	854 ± 83**
**TAU**	298 ± 38*	3.5 ± 0.5**	48 ± 6**	1258 ± 141**

Data are expressed as mean ± S.E.M. of 9 rats. *P < 0.05 and **P < 0.01 compared to rats injected with saline (SAL). # *P *< 0.05 compared with PLA_2 _group.

### Concentration-response curves to vasodilator agents

**Figure 1:**
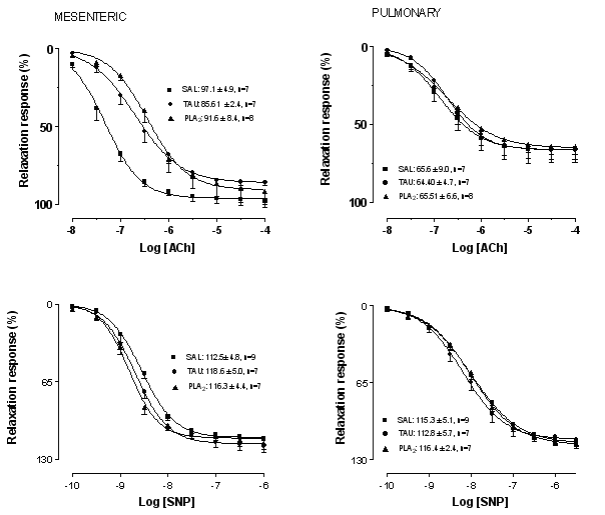
**Concentration-response curves to acetylcholine (ACh, panel A) and sodium nitroprusside (SNP, panel B) in mesenteric and pulmonary rings from ■ Saline (SAL), ● taurocholate (TAU), and ▲ phospholipase A2 (PLA2)**. Maximal responses values were inserted in the figure. Data are mean ± SEM for 7–8 animals.

Acetylcholine (ACh) produced a concentration-dependent relaxing response in both mesenteric and pulmonary rings. In mesenteric rings, the potency for ACh was significantly decreased from animals treated with TAU (4.2-fold) and PLA_2 _(6.9-fold) compared to saline group. No changes were seen in the maximal responses for the muscarinic agonist in all groups (Table 2 and Figure 1). In pulmonary rings, neither potency nor maximal responses for ACh were altered in any groups (Table 2 and Figure 1).

**Table 2:** Potency of acetylcholine (ACh), sodium nitroprusside (SNP) and phenylephrine (PHE) from rat arteries after pancreatitis

	**Mesenteric Artery**			**Pulmonary Artery**		
		
**Groups**	**ACh**	**SNP**	**PHE**	**ACh**	**SNP**	**PHE**
**SAL**	7.26 ± 0.06	8.58 ± 0.07	6.88 ± 0.06	6.86 ± 0.07	8.02 ± 0.05	7.00 ± 0.09
**TAU**	6.64 ± 0.14*	8.67 ± 0.06	6.54 ± 0.09*	6.66 ± 0.8	8.18 ± 0.08	6.57 ± 0.05*
**PLA**_2_	6.42 ± 0.08*	8.79 ± 0.04	6.74 ± 0.12	6.69 ± 0.10	8.02 ± 0.09	6.92 ± 0.07

Potency (pEC_50_) of acetylcholine (ACh), sodium nitroprusside (SNP) and phenylephrine (PHE) obtained from concentration-response curves in mesenteric and pulmonary rings from saline (SAL); taurocholate (TAU) and phospholipase A_2 _(PLA_2_) groups. Potency is represented as -log of the molar concentration to produce 50% of the maximal response. Data are mean ± SEM for 5–9 animals. * different from SAL (*P *< 0.05).

The nitric oxide (NO) donor, sodium nitroprusside (SNP) produced a concentration-dependent relaxing response in both mesenteric and pulmonary rings. The potency and the maximal responses for SNP were not changed in rat mesenteric and pulmonary rings in all groups (Table 2 and Figure 1).

### Concentration-response curves to vasoconstrictor agent

**Figure 2:**
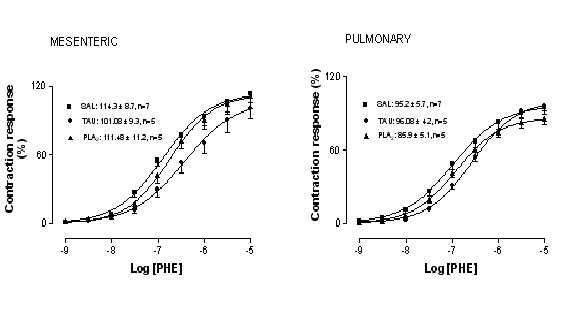
**Concentration-response curves to phenylephrine (PHE) in mesenteric and pulmonary rings from ■ Saline (SAL), ● taurocholate (TAU), and ▲ phospholipase A_2 _(PLA_2_)**. Maximal responses values are inserted in the figure. Data are mean ± SEM for 7 animals.

Phenylephrine produced concentration-dependent contractile responses in both mesenteric and pulmonary rings in all groups. The potency for this α-adrenergic agonist was significantly decreased in rat mesenteric and pulmonary rings from TAU group compared to SAL group (about 2.2- and 2.7-fold for mesenteric and pulmonary rings, respectively, Table 2). No changes were seen in the maximal responses for the α-adrenergic agonist in this pancreatitis model (Figure 2). Neither potency nor maximal responses for PHE were affected by PLA_2_-induced pancreatitis (Table 2 and Figure 2).

### Serum nitrite/nitrate (NO_x_^-^) levels

The nitrite/nitrate (NO_x_^-^) levels were markedly increased in animals submitted to pancreatitis (TAU: 23.2 ± 2.4 and PLA_2_: 21.8 ± 2.4 μM) when compared to SAL group (13.0 ± 1.5 μM, *P *< 0.05), approximately 76 and 68% in TAU and PLA_2_, respectively.

## Discussion

The present study is the first to examine the vascular reactivity of isolated mesenteric and pulmonary rings after acute pancreatitis in rats. Our findings show that acute pancreatitis promotes reduction in the potency for ACh in mesenteric artery that was accompanied by marked increase in nitrite/nitrate levels. Decreased sensitivity of contracting response for PHE in both mesenteric and pulmonary preparations was also found.

Injection of PLA_2_s [11,12] and taurocholate [10,13,14] into the common bile duct has been used as an useful model to reproduce the local and remote inflammation observed in the human pancreatitis, as observed by the increased vascular permeability and neutrophil influx in the pancreas that is accompanied by elevated serum amylase levels and neutrophil accumulation into the lungs. Systemic haemodynamic disturbances are important complications of pancreatitis either in animal models [9] or in human [15]. Evidences show that arterial hypotension seen in pancreatitis has been associated with a massive overproduction of NO [9,16,17]. Our data show a significant decrease in endothelium-dependent relaxation response in mesenteric artery from both TAU and PLA_2 _acute pancreatitis without changing for SNP. These findings suggest that both pancreatitis conditions lead to an endothelial dysfunction in mesenteric rings. Indeed, a marked increase in NOx^- ^levels (approximately of 68–76%) in both experimental pancreatitis models was seen in our experimental protocol. Several lines of evidence have pointed out that certain disorders such as atherosclerosis, diabetes mellitus and inflammatory diseases provoke severe tissues damage and endothelial dysfunction that is strongly linked to overproduction of NO derived from leukocytes and/or reduction in the activity of antioxidant system leading to a massive formation of reactive species of oxygen [15,17-21]. Our findings show clearly a strong relationship between NOx^-^levels and decrease in endothelium-dependent relaxation response in mesenteric artery suggesting that the intense inflammatory responses in response to acute experimental pancreatitis promote severe arterial endothelial damage. On the other hand, no changes were seen in relaxing response from pulmonary rings. The reason for that could be the time elapsed after acute pancreatitis, four hours. Thus, the endothelial cells from pulmonary arteries were not exposed to the massive production of reactive species of oxygen produced by pancreatitis in its early phase. Furthermore, previous study reported that the up-regulation of inducible NOS (iNOS) reach its maximum expression about six to twelve hours after pancreatitis induction [16].

Regarding to contractile response, TAU-induced pancreatitis caused a reduction of potency for PHE in both mesenteric and pulmonary artery rings, but no changes were found in contracting responses from PLA_2 _group. Although it could be argued that an overproduction of NO could explain such discrepancy, our data that the levels of NOx^- ^were similar in both groups exclude this possibility. It is well-established that mortality of rats in TAU-induced acute pancreatitis is higher compared to other pancreatitis models [22,23]. Indeed, TAU has been shown to induce a severe form of pancreatitis since it induces haemorragic necrotizing effects in the pancreas associated with higher remote inflammation in rats [10], which was confirmed in the present study. Thus, the subsensitivity of contractile response for PHE in isolated mesenteric and pulmonary rings from TAU-induced pancreatitis seen in this experimental model may reflect extensive cell damage secondary to massive systemic enzyme generation (amylase, lipase and others).

## Conclusion

In conclusion, the acute pancreatitis provoked deleterious effects in endothelium-dependent relaxing response for ACh in isolated mesenteric rings that were strongly associated with high plasma NO_x_^- ^levels as consequence of intense inflammatory responses. Furthermore, the subsensitivity of contractile response to PHE in both mesenteric and pulmonary rings might be due to the complications of this pathological condition in the early stage of pancreatitis.

## Abbreviations

NO: nitric oxide; SAL: saline; TAU: tauracholate; PLA_2_: phospholipase A_2_; ACh: acetylcholine; SNP: sodium nitroprusside; PHE: phenylephrine; pEC_50 _: potency; E_MAX _: maximal response; NO_x_^- ^: nitrite/nitrate; iNOS: inducible nitric oxide synthase.

## Competing interests

The authors declare that they have no competing interests.

## Authors' contributions

EAC and MAD carried out the acute pancreatitis induction, NO_x_^- ^determinations, manuscript preparation; and the concentration-response curves; TF helped the NO_x_^- ^determinations and acute pancreatitis induction; ECTL participated in the design of the PLA_2_-induced pancreatitis model; EA and AZ participated in the study coordination, in the preparation and revision of the manuscript. All authors read and approved the final manuscript

## Pre-publication history

The pre-publication history for this paper can be accessed here:


